# The use of health facility data to assess the effects of armed conflicts on maternal and child health: experience from the Kivu, DR Congo

**DOI:** 10.1186/s12913-021-06143-7

**Published:** 2021-09-13

**Authors:** Espoir Bwenge Malembaka, Chiara Altare, Rosine Nshobole Bigirinama, Ghislain Bisimwa, Robert Banywesize, Nabil Tabbal, Ties Boerma

**Affiliations:** 1 Ecole Régionale de Santé Publique (ERSP), Faculté de Médecine, Université Catholique de Bukavu, Bukavu, DR Congo; 2 Institute of Health and Society (IRSS), Université Catholique de Louvain, Brussels, Belgium; 3 Johns Hopkins Bloomberg School of Public Health, Johns Hopkins University, Baltimore, USA; 4 Division Provinciale de la Santé du Sud-Kivu, Ministère Provincial de Santé Publique, Bukavu, DR Congo; 5 World Health Organization, Geneva, Switzerland; 6 Centre for Global Public Health, Department of Community Health Sciences, Rady Faculty of Health Sciences University of Manitoba, Winnipeg, Manitoba Canada

**Keywords:** Health facility data, DHIS2, Maternal and child health, Conflict, DRC

## Abstract

**Background:**

In conflict-affected settings, data on reproductive, maternal, newborn and child health (RMNCH) are often lacking for priority setting and timely decision-making. We aimed to describe the levels and trends in RMNCH indicators within Kivu provinces between 2015 and 2018, by linking conflict data with health facility (HF) data from the District Health Information System 2 (DHIS2).

**Methods:**

We used data from the DHIS2 for the period 2015–2018, the 2014 Demographic and Health Survey, the 2018 Multiple Indicators Cluster Survey and the Uppsala Conflict Data Program. Health zones were categorised in low, moderate and high conflict intensity level, based on an annual conflict death rate. We additionally defined a monthly conflict death rate and a conflict event-days rate as measures of conflict intensity and insecurity. Outcomes were completion of four antenatal care visits, health facility deliveries, caesarean sections and pentavalent vaccine coverage. We assessed data quality and analyzed coverage and trends in RMNCH indicators graphically, by conflict categories and using HF data aggregated annually. We used a series of fixed-effect regression models to examine the potential dose-response effect of varying conflict intensity and insecurity on RMNCH.

**Results:**

The overall HF reporting was good, ranging between 83.3 and 93.2% and tending to be lower in health zones with high conflict intensity in 2016 and 2017 before converging in 2018. Despite the increasing number of conflict-affected health zones over time, more in North-Kivu than in South-Kivu, we could not identify any clear pattern of variation in RMNCH coverage both by conflict intensity and insecurity. North-Kivu province had consistently reported better RMNCH indicators than South-Kivu, despite being more affected by conflict. The Kivu as a whole recorded higher coverage than the national level. Coverage of RMNCH services calculated from HF data was consistent with population-based surveys, despite year-to-year fluctuation among health zones and across conflict-intensity categories.

**Conclusions:**

Although good in general, the HF reporting rate in the Kivu was negatively impacted by conflict intensity especially at the beginning of the DHIS2’s rolling-up. Routine HF data appeared useful for assessing and monitoring trends in RMNCH service coverage, including in areas with high-intensity conflict.

**Supplementary Information:**

The online version contains supplementary material available at 10.1186/s12913-021-06143-7.

## Background

Akin to many remote and conflict-affected settings, empirical data on reproductive, maternal, newborn and child health (RMNCH) are often lacking for priority setting, timely decision-making and resource allocation in the Kivu provinces in the Democratic Republic of the Congo (DRC). Limited access to affected areas as well as insufficient human, technical and financial resources for health information systems at district or operational level lead to high variability in terms of coverage, functionality and quality of collected data. Nationally representative surveys such as Demographic and Health surveys (DHS) or Multiple Indicator Cluster Surveys (MICS) are key to document long-term trends in health indicators in low- and middle-income countries. Recent analyses of household surveys confirmed the destructive effects of armed conflicts on women’s and children’s health [1–3]. However, the design of these surveys generally does not allow for disaggregation beyond the provincial or regional level or for short-term trend analysis of RMNCH interventions coverage. This would be particularly important in humanitarian emergencies where the situation evolves rapidly.

The health system in DRC has been weakened by decades of protracted socio-political turmoil and conflict (Panel). The DRC generally performs poorly on RMNCH indicators with maternal mortality ratio and under five mortality rate estimated in 2017 at 473 per 100,000 [4] and 88 per 1000 live births in 2018 respectively [5]. Over half of children under 5 years of age are stunted in the Kivu [6]. The universal health coverage index in DRC was 40% in 2015, far below the global median (65%) in the WHO member states [7]. Some reports have suggested mitigated effects of conflict on maternal and child health indicators in eastern DRC provinces [2, 8] where the health system is claimed to be the best funded of the country, owing substantially to humanitarian aid [9]. But, generally, attempts to assess the impact of conflict on RMNCH coverage indicators in these provinces have been hampered by lack of data.

The quest for context-sensitive data sources in conflict settings has justified the exploration of various alternatives (from small scale surveys, community-based surveillance, to Early Warning, Alert and Response System), with variable results [10–15]. Strengthening health facility data management at health zone (HZ) or district level is likely one of the more sustainable strategies to ensure widespread coverage and needed granularity of information [16]. Well characterised health facility data are likely to provide crucial disaggregated and potentially timely information for program planning and resource allocation at small geographical level in conflict settings that complement nationwide population-based surveys conducted on average every 5 years [17, 18]. However, concerns so far have been raised about data completeness and accuracy as well as the difficulties of calculating population denominators.

Reaching all women and children with RMNCH interventions is central in the health Sustainable Development Goals. Innovative approaches to measuring progress in RMNCH are needed to document whether and why vulnerable people in conflict-affected settings are being left behind [19–22]. This study aimed to describe the levels and trends in RMNCH indicators by health zone within Kivu provinces between 2015 and 2018, by linking conflict data with health facility data from the District Health Information System 2 (DHIS2).

### Panel: protracted conflict and health system structure in DRC

**Table:** 

Eastern DRC has been plagued by decades of socio-political instability and insecurity, despite the official end of the war in December 2002 with the Pretoria agreement [23]. North and South Kivu provinces stretch over 114,274 km^2^ sharing borders with Burundi, Rwanda and Uganda. These natural resource-rich provinces have been characterized by conflicts over land, power and ethnicity since the pre-colonial epoch [24, 25]. Fourteen years after 2006 first DRC presidential election [26], more than 140 armed groups are operating in the Kivu provinces [27], many of them fighting for control over natural resources sold largely to the international community [28]. The Kivu was home to about 16.03 million inhabitants in 2018 of which 8.93 (55.7%) were in North Kivu, according to population estimates used by the ministry of health and available in the DHIS2. There were 5.01 million internally displaced persons recorded between January 2018 and December 2019, of with 20.1% (about million) located in North Kivu and 17% (847.5 thousands) in South Kivu. Over half (50.9%) of IDPs were women and 57.9% were children [29]. There are IDPs settlements in the Kivu [30], but information on their number and size is sparse. The Uppsala Conflict Data Program (UCDP) [31, 32] has investigated the DRC conflict since 1989 and has documented 112,220 direct conflict fatalities for the period 1989-2018, 60% of which occurred in the Kivu. An analysis of the Kivu Security tracker reports (*https://kivusecurity.org/reports*) between October 2017 and December 2019 recounts a monthly average of 84 violent deaths, 50 clashes between belligerents and 122 abductions and kidnappings in the Kivu. Some bursts of killings of civilians by the Ugandan Allied Democratic Forces–NALU in Beni (North Kivu) have been associated with Islamist claims [33], further complicating the conflict puzzle. In general, North Kivu has experienced higher conflict intensity than South Kivu, however variation in terms of conflict events and deaths is high across health zones. Frequent eruptions of ethnic violence have worsened the already dire crisis in a country that had over 4.5 million internally displaced persons, the second highest number after Syria (as of end of 2018) [34]. In DRC, the health system structure comprises two operational levels within provinces. The primary healthcare services are organised around health centres providing the minimum service package. This includes family planning, antenatal- and postnatal care, obstetric care (for normal deliveries), newborn care, child vaccination, integrated management of childhood illness, and treatment of severe acute malnutrition. The second level is centred around HZ hospitals offering a complementary package that covers internal medicine, hospitalisation, surgical and reference services, in addition to technical support to health centres through integrated supervision [35]. As of 2017, there are 68 health zones in the Kivu (34 in each province). There are 2079 health facilities in the Kivu, of which 50% are public, 37% are owned by faith-based organisations, and 13.4% are private.	

## Methods

### Data sources

As in many low and middle-income countries, routine health facility data are recorded on paper, compiled through the web-based District Health Information System 2 (DHIS2) at the health zone level and uploaded to the provincial health division headquarters on a monthly basis [36]. The DHIS2 was first introduced in DRC in 2012 and scaled up to South-Kivu and North-Kivu provinces as of 2015. The DHIS2 is used by all public, and the majority faith-based and private health facilities in the Kivu.

This study used monthly health facility data available via the DHIS2, disaggregated at health zone level and covering the period January 2015 to December 2018. We also used publicly available reports of the 2014 DHS [37] and 2018 MICS [38] to ascertain the levels and trends in RMNCH indicators at the provincial and national levels. One of the 32 clusters targeted in North-Kivu in the 2014 DHS was not accessible due to insecurity. Similarly, one of the 27 clusters targeted in the 2018 MICS was not visited. All clusters in South-Kivu were accessible. Within the visited clusters response rates were high. Therefore, the effect of non-response due to insecurity on the survey results is likely to be small.

Conflict data were retrieved from the Uppsala Conflict Data Program Georeferenced Event Dataset (UCDP GED) [31] for the period 2015–2018. UCDP GED provides publicly available data on conflict events, deaths, actors, dates and locations. Data related to organized violence across Africa are georeferenced since 1989. The use of the UCDP data in conflict epidemiology has intensified over the last decade, because of their higher quality and reliability compared to other conflict data sources, particularly when the focus is on subnational level analysis of conflict [31, 39]. Health zones shape files were obtained from the United Nations Office for Coordination of Humanitarian Affairs (UNOCHA) [40].

### Data process

We followed a step-wise approach to assessing health facility data quality as recommended by the World Health Organization [41, 42] and applied the method for computation of coverage indicators using health facility data used by Maina and colleagues in Kenya [41]. This comprises an analysis of four data quality dimensions: completeness of reporting, internal consistency over time, external consistency with related indicators and external comparison with population data. A full assessment of the data quality metrics is presented in the additional file. Here, we present only results on reporting completeness which we deemed the most reliable dimension as less affected by service disruption due to conflict. Reporting completeness is defined as the percentage of expected reports from health facilities that have been actually received at provincial level. Before calculating the coverage of RMNCH services, we checked and corrected extremely implausible reported numbers and adjusted for incomplete reporting by health facilities.

We calculated modified Z-score, as proposed by WHO, to identify extremely implausible reported annual values for the selected indicators [43]. The modified Z is an approximation of the difference of a score from the median value and was calculated as:

1 $$ {M}_i=\frac{0.6745\times \left({x}_i-{x}^{\sim }\ \right)}{MAD} $$

where: x_i_ denotes the value of the observation for a particular year, x ~ is the median of the series (48 months, from January 2015 to December 2018), and MAD is the median absolute deviation (MAD = median (|x_*i*_ - x ~ |).^1^

We considered a modified Z greater than five as indicative of a potential extreme outlier [43]. Given that, in conflict settings, the provision of RMNCH health services may significantly fluctuate as a result of conflict, making the correction of outliers tricky. Our correction strategy was therefore conservative. An extreme outlier (modified Z > 5) was deemed implausible and corrected if the reported number was 3 times greater than the expected value. Details about the estimation of the expected numbers are presented in the Additional file.

The adjustment for incomplete reporting was guided by the assumptions about volume of services provided by the non-reporting facilities. Each service coverage was calculated at health zone level by adjusting the numerators as follows:

2 $$ n(adjusted)=n+n\left(\frac{1}{c}-1\right)\ast k $$

where *n* is the number of service outputs as per DHIS2; *c* is the reporting completeness, *k* is the adjustment factor varying between 0 and 1 depending on the assumption made on the level of service provision in non-reporting heath facilities compared to reporting health facilities [41]. We considered that in conflict-affected settings, no reporting could be reflective of unavailability of service. We therefore selected a small value of k (0.25), assuming some level of service delivery in non-reporting health facilities, but much lower than in reporting health facilities. We conducted sensitivity analyses with k values of 0 and 1, assuming no service provision in non-reporting health facilities or service provision to the same level as reporting health facilities, respectively (Additional file). The adjustment for reporting incompleteness was used for the analysis based on annually aggregated data because information about reporting rate was not accessible for monthly disaggregated data.

The latest population census in DRC dates back to 1984, making it challenging to define accurate target populations for health interventions. We obtained the total population estimates for HZ from the DHIS2. The DRC Ministry of Health (MoH) updates its population estimates by applying a population growth rate of 3%. On occasion, local population counts are conducted at HZ or lower level for specific health programs. For example, the South-Kivu MoH carried out a population count in 2014 prior to a mosquito nets distribution campaign. However, such population counts are often subject to under- or over-reporting biases [44].

We derived specific target populations from the first antenatal care (ANC) visit which had a near-universal coverage in the Kivu according to the 2018 DRC MICS [38]. The number of pregnancies was obtained by adding to the reported ANC number in the DHSI2 2.5 and 6.5% of pregnant women not attending even one ANC visit in North-Kivu and South-Kivu respectively [6]. We subtracted the proportion of pregnancies ending in early foetal death (here defined as before the first ANC visit, 5%) to obtain the number of deliveries. We added 2% of multiple pregnancies to obtain the number of births [45]. For live births, we subtracted the proportion of stillbirths (2%) [46]. For the number of infants targeted for immunisation, we subtracted the proportion of newborns who died in the first month of life (2.8%) from the number of live births [47].

### Definition of the conflict exposure variable

In conflict epidemiology, there is no one-size-fits-all definition of exposure to conflict and different authors have used various definitions depending on context, study design and objectives [1, 2, 48–50]. We considered armed conflict from two angles: conflict events and deaths, in order to capture both the direct impact of armed conflict on populations (deaths) as well as the instability caused by recurrent conflict events (independently of direct fatalities) at the health zone level. We created three variables. An annual conflict death rate at HZ level was defined as the annual cumulative number of conflict-related fatalities divided by the population estimate for the same year. As no standard threshold exists, we considered the distribution of deaths and classified the conflict intensity in HZ as low, moderate and high based on an annual death rate below 1, between 1 and 4.9, and above 5 per 100,000 inhabitants respectively. We explored other possibilities for classifying HZ in terms of conflict intensity (Additional file). However, the classification based on annual conflict death rate appeared to echo better the perceived situation on the ground.

As the annual conflict death rate may fail to capture variation in conflict events over a shorter time frame, we developed two additional conflict measures using monthly data. A monthly conflict death rate at HZ level was calculated as the cumulative number of deaths in each month and HZ per 100,000 population. We also approximated a monthly insecurity rate at HZ level by a conflict event-days rate per 100,000 population. This was calculated as the cumulative number of days from events that occurred during a given month and in each health zone and normalized for its population. In the situation where a conflict event spans over 2 months or more, the number of days occurring in a specific month are taking into account for that month.

#### Using the monthly health facility data to examine the impact of armed conflict on RMNCH service

Our analysis of the impact of conflict on RMNCH focused on key service indicators along the RMNCH continuum: completion of four antenatal care visits (ANC4), health facility deliveries, health facility caesarean sections (CS), and third dose of the pentavalent vaccine. Indicators definitions are presented in the Additional file. We assessed the level and consistency of the coverage estimates based on the annual data from the DHIS2 with the health surveys, particularly the MICS 2018, for North-Kivu and South-Kivu. Since the surveys collect retrospective data we time located the RMNCH coverage estimates from the MICS 2018 in 2017 (as MICS provides two-year rates) and the DHS 2014 in 2012 (as DHS generally provides 5 year rates for MNH indicators, except for immunisation data that corrects children aged 12–23 months in the year before the survey). We then conducted descriptive analysis of the levels and trends in RMNCH coverage indicators graphically, stratified by the levels of the annual conflict death rates and using health facility data aggregated annually.

Next, we examined the relationship between conflict intensity and insecurity, and RMNCH service provision indicators using monthly data. We carried out graphical and statistical analyses of the difference between reported and expected numbers of RMNCH indicators if there was no conflict in a certain health zone. We first calculated the average value of the health indicators for all the non-conflict months. We considered this average as the expected value for the middle month of the entire time series (that is, January 2017). We computed a monthly growth rate as one-twelfth of 3% of the reported number, assuming a 3% annual increase. The growth rate was then added to and subtracted from the expected central value to estimate expected number for the next and previous months, respectively. The same process was repeated for each of the four indicators. We used both reported and expected numbers to calculate the reported and expected rates respectively.

We graphically examined the difference between reported numbers during severe conflict and mild or non-conflict months relative to the estimated expected numbers if there was no conflict (Additional file). We then used a series of fixed-effect regression models with robust standard errors to further examine the effect of conflict on RMNCH. The outcome was the difference between reported and expected rates of the indicator. We examined the dose-response effect of varying levels of conflict intensity and insecurity on RMNCH. We successively run three regression models with binary conflict intensity and insecurity variables defined by monthly fatality rate at least equal to 5, 10 and 15 deaths per 100,000; and an event-day rate at least equal to 1, 5 and 15 conflict events per 100,000 population respectively. Fixed-effect models were chosen to control for all the unmeasured time-invariant differences between the health zones like local socio-cultural norms, religion, gender inequality, maternal literacy, ethnicity, socio-economic development and rurality.

Two series of regression models were built to examine 1) the effect of a contemporaneous armed conflict on RMNCH service provision, 2) a one-month lagged effect of conflict intensity and insecurity on RMNCH service provision. We considered a *p* value < 0.05 as statistically significant. We used QGIS 3.4 software to map conflict data by health zone and to ease their merging with health facility data. Statistical analyses were done with Microsoft Excel 2017 and Stata 16.

## Results

### Conflict intensity

**Fig. 1:**
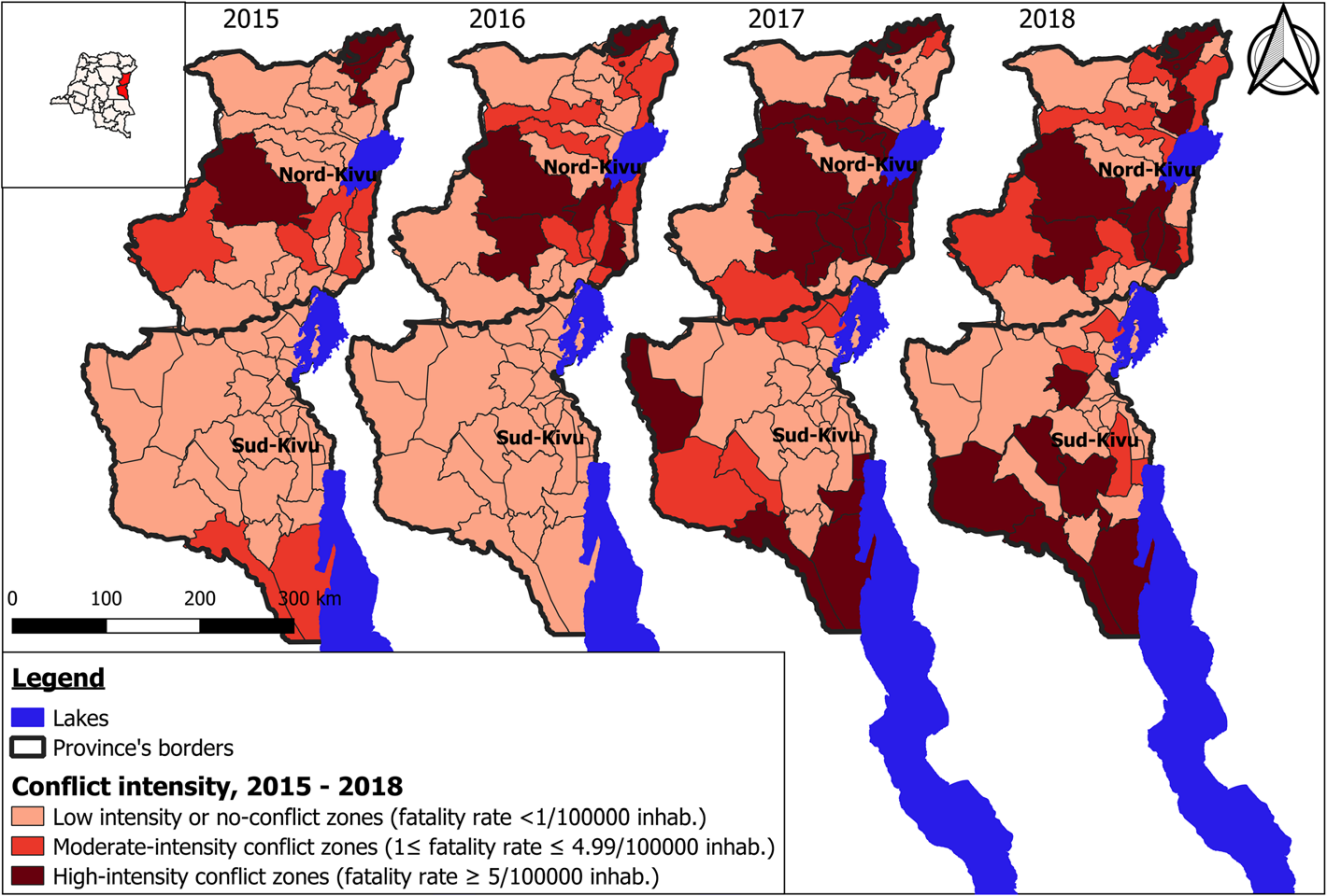
Classification of Kivu’s health zones by conflict intensity (as per annual death rate), 2015–2018

Overall, conflict seemed consistently more intense in North-Kivu than in South-Kivu and increased over time (Fig. 1). There was great variability in conflict intensity between HZ and over time. The number of HZ experiencing moderate to high-intensity conflict rose from 11 (16.2%) in 2015 to 29 (44.7%) in 2018 (Table S2 and Fig. S2, Additional file).

### Reporting completeness

**Fig. 2:**
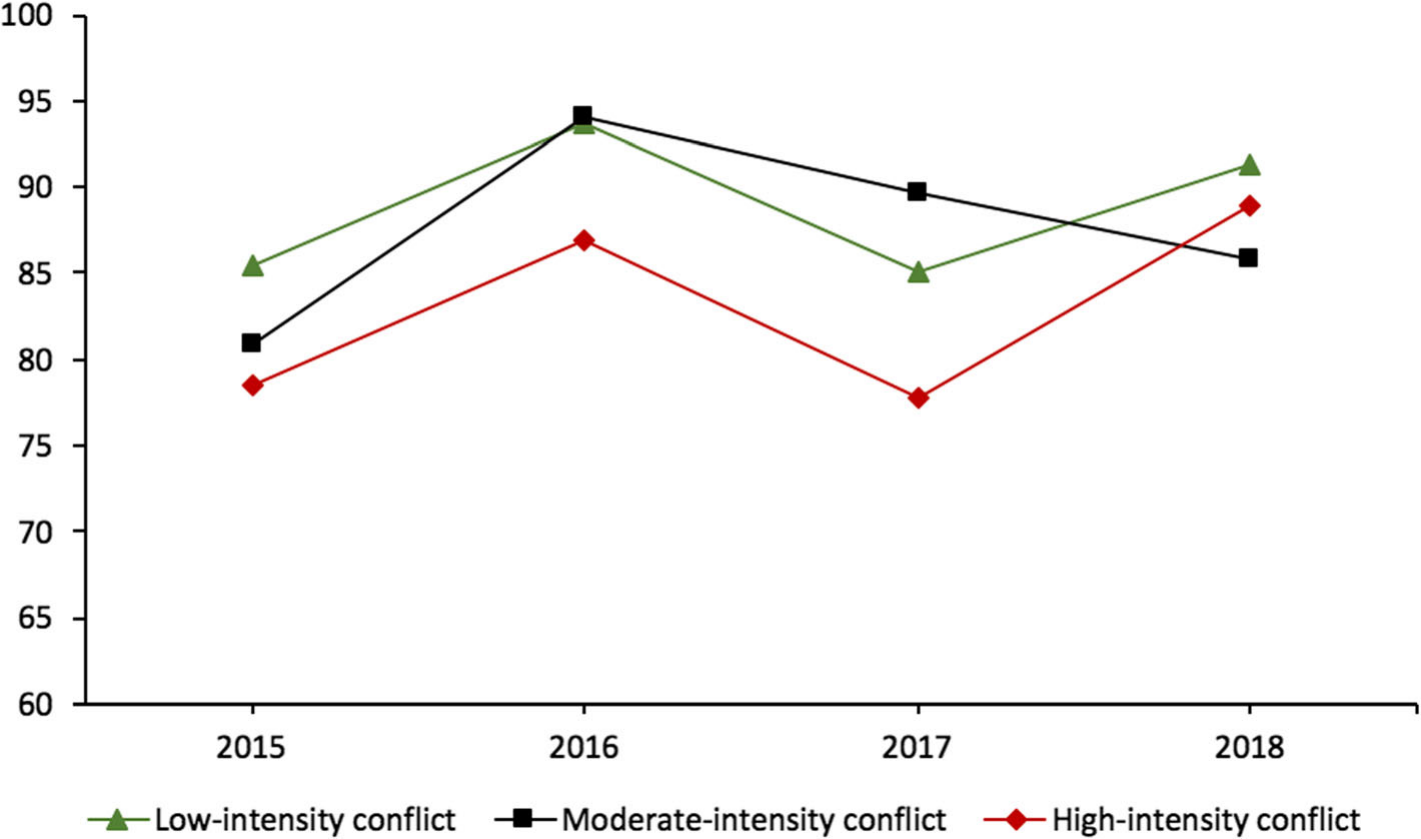
Reporting completeness in the Kivu, by conflict intensity (as per annual death rate), 2015–2018

Health facility reporting completeness ranged between 83.3% (in 2017) and 93.2% (in 2016) in the Kivu, and was on average higher in South-Kivu (91.4%) than in North-Kivu (84.3%). HZ with high conflict intensity to had a 10–15% lower reporting rate than the HZs less affected by conflict, especially in 2016 and 2017 (Fig. 2).

### Levels and trends in key RMNCH in the Kivu provinces

**Fig. 3:**
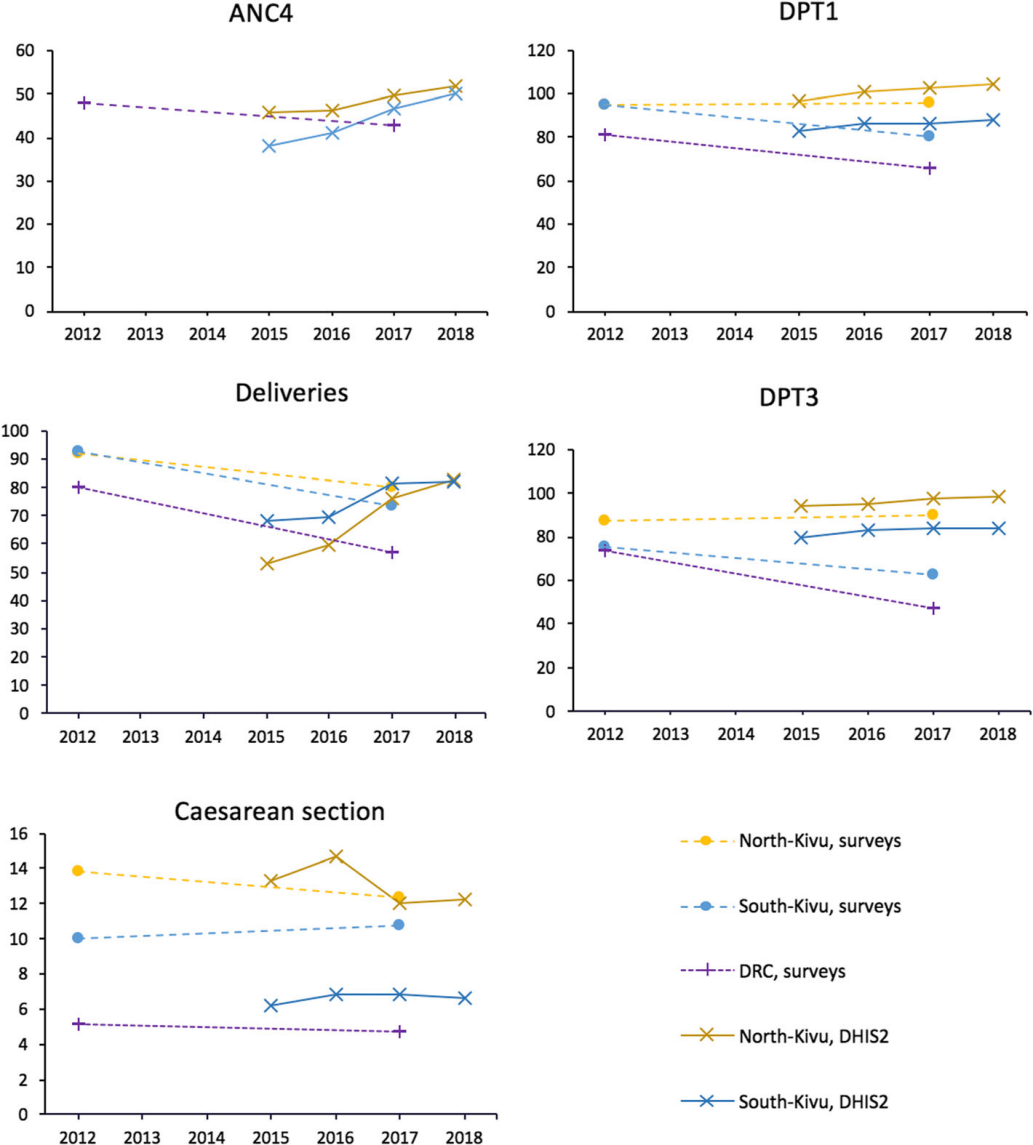
Trends in RMNCH indicators in North-Kivu and South-Kivu, 2012–2018. Note: No estimate for ANC4 at provincial level was available in the 2014 DRC DHS. According to the 2018 DRC MICS report, the ANC4 coverage in North-Kivu and South-Kivu was 55.9 and 46.3% respectively, and 42.9% nation-wide

Figure 3 describes the levels and trends in antenatal care, proportion of facility deliveries, caesarean section and pentavalent vaccine coverage in North-Kivu and South-Kivu. The levels of RMNCH indicators were higher in North-Kivu than in South-Kivu and both provinces had higher levels than national averages (Fig. 3). The proportion of pregnant women completing ANC4 was consistent with survey data and increased over time in all conflict intensity categories. The proportion of deliveries in health facilities in the DHIS2 was also in agreement with surveys results and tended to be higher than the national average without any notable variation by conflict intensity. National surveys showed a decreasing trend in health facility deliveries, which is not reflected in DHIS2 data.

**Fig. 4:**
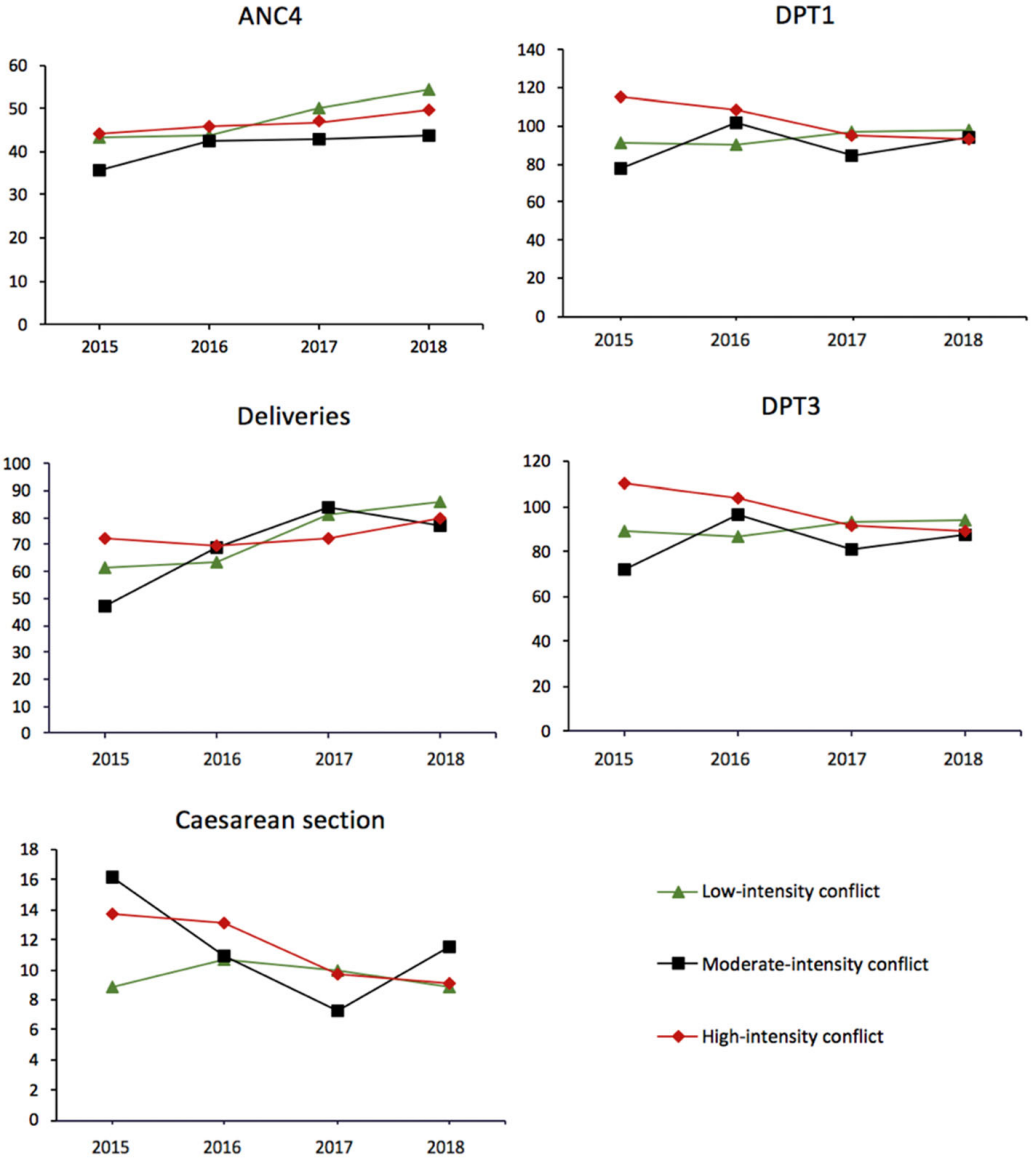
Trends in RMNCH indicators by level of conflict intensity in selected health zones, 2015–2018

Health zones with high-intensity conflict seemed to conduct more caesarean sections in 2015 and 2016 than those with lower conflict intensity, but the gap tended to close from 2016 on. The level of caesarean section in the Kivu (regardless of conflict intensity level) was higher than the national average. The trends in the coverage of the first and third doses of the pentavalent vaccine was almost identical, with overestimated coverage in HZ with high-intensity conflict in 2015 and 2016 and generally better in the Kivu than countrywide (Fig. 4).

Results of the regression analysis indicate statistically insignificant contemporaneous (Table 1) and lag effects (Table S10, Additional file) of conflict intensity and insecurity on RMNCH service provision insecurity (Table 1).

**Table 1:** Regression analysis of the effects of conflict intensity and insecurity on RMCH service provision in the Kivu

Outcome	Threshold 1		Threshold 2		Threshold 3	
Difference, reported-expected numbers	Coefficient (95% CI)	*P* value	Coefficient (95% CI)	*P* value	Coefficient (95% CI)	*P* value
ANC4						
Severe conflict	1.79 (−1.30; 4.88)	0.251	1.49 (−1.67; 4.64)	0.354	1.27 (−1.94; 4.48)	0.432
Insecurity	1.26 (−1.19; 3.71)	0.309	1.50 (−1.55; 4.54)	0.329	−0.44 (− 4.22; 3.34)	0.818
Facility deliveries						
Severe conflict	2.99 (−2.39; 8.37)	0.271	3.43 (− 2.74; 9.59)	0.271	3.69 (− 2.03; 9.40)	0.202
Insecurity	3.04 (−1.89; 7.97)	0.222	2.36 (−3.32; 8;03)	0.410	2.84 (−7.42; 13.09)	0.583
Caesarean section						
Severe conflict	0.11 (−0.49; 0.71)	0.721	0.01 (−0.63; 0.65)	0.980	0.03 (−0.56; 0.62)	0.915
Insecurity	0.07 (−0.47; 0.61)	0.800	0.20 (−0.39; 0.79)	0.508	−0.23 (−1.09; 0.64)	0.604
DPT3						
Severe conflict	1.35 (−1.67; 4.37)	0.375	0.53 (−2.91; 3.97)	0.760	0.21 (−3.17; 3.58)	0.903
Insecurity	1.45 (−1.28; 4.19)	0.293	1.86 (−1.19; 4.91)	0.227	−0.39 (−5.32; 4.54)	0.874

Threshold 1: Severe conflict = monthly conflict death rate ≥ 5 per 100,000 population; insecurity = conflict event-days ≥1 per 100,000 population. Threshold 2: Severe conflict = monthly conflict death rate ≥ 10 per 100,000 population; insecurity = conflict event-days ≥5 per 100,000 population. Threshold 3: Severe conflict = monthly conflict death rate ≥ 15 per 100,000 population; insecurity = conflict event-days ≥10 per 100,000 population

## Discussion

This study explores the usefulness of monthly health facility data in measuring progress in RMNCH service provision while taking into account the ongoing armed conflict in the Kivu provinces of DRC. Despite the increasing number of conflict affected HZs over the study period [51], the effect of conflict on RMNCH service provision is not straightforward to measure and assess. North-Kivu province has consistently reported better RMNCH indicators than South Kivu, despite being markedly more affected by conflict. The Kivu province as a whole recorded higher coverage than the national level. Completeness of reporting seemed however affected by conflict in 2016–2017 but has converged to higher levels in 2018.

Overall, the levels of antenatal care, health facility deliveries, child immunisation coverage and caesarean sections calculated from health facility data were consistent with those reported in population-based surveys, despite year-to-year fluctuation among HZ and across conflict-intensity categories. These finding points to a successful roll-out and scale-up of DHIS2 in these conflict-torn provinces, which enables the district to have a quite reliable assessment of the situation in the Kivu provinces, although with some initial challenges. Health facility reporting tended to be poorer in HZ with higher conflict intensity in 2016–2017, possibly highlighting the complexity of rolling out a new information system in conflict-affected areas. Over time however, health facilities, district and provincial offices seem to have found ways to make such reporting possible. A functioning health information system requires skilled and motivated personnel, access to basic information and communication technologies, electricity and roads. All these factors are likely to be negatively impacted by armed conflict, through direct attacks and looting as well as indirectly due to insecurity, reduced mobility, and limited availability of financial and human resources [52]. Strategies to improve routine facility data gathering and reporting that are tailored to conflict affected settings can contribute to improve the effectiveness of the system. In DRC, paper-based reports from health facilities are captured into the DHIS2 at the HZ headquarters. When reports from health facilities cannot reach the HZ headquarters because of active conflict or impassable roads, the use of mobile phone technologies for data entry at the facility level could represent an alternative mechanism for data sharing. Such an option seems becoming more and more feasible thanks to both the increasing cellular network coverage in many parts of the DRC (although not yet complete) [53], and the possibility for offline data capture now available within DHIS2 mobile application [54]. While this is not currently used in DRC, it represents one of the next investments that would build upon the results achieved so far. This would also contribute to improve the timeliness of reporting, another key feature of routine data. Lags of several weeks are not uncommon, restricting therefore the potential for such data to inform real time decision making.

In line with a few other studies on eastern DRC, our selection of key maternal and child health indicators reported higher levels in these conflict-affected provinces than in the rest of the country [2, 8, 55, 56]. Some factors may contribute to explain this pattern. First, the poverty level as measured by the latest MICS seems to be higher in western and central DRC provinces than in the Kivu [38]. Users’ fees, as applied in DRC, may therefore represent an important barrier to access to health services. Second, the health system in eastern DRC provinces is reportedly better funded than in other provinces, mainly thanks to humanitarian aid [57, 58]. In some conflict-affected HZ, RMNCH services are totally subsidised by non-governmental organisations, often waiving users’ fees. This may result in improvements in both access to health services and retention of healthcare workers through payment of wages and incentives [59]. Third, many humanitarian agencies are financially supporting HZ under a performance-based contracting scheme [60], with clear emphasis on health facility reporting [61]. This has likely contributed to reinforcing the culture of monitoring and reporting in these provinces. Fourth, communities and service providers might have learned to adapt to a situation of chronic conflict characterised by a multiplicity of armed groups based in remote areas and intermittently disrupting lives and services [62]. Unstable and varying alliances along ethnic lines coupled with political and financial interests make the conflict dynamics highly volatile [63].

This form of conflict results in high level of unpredictability, requiring high adaptation capacity, negotiation skills as well as context-adapted solutions. At the same time, it also allows for certain health services to continue during moments of calm. Schedulable services, such as preventive services may therefore seem less affected. This could be the reason why we were not able to identify variations in ANC4 or DPT1. More surprisingly were the rather stable trends in both facility deliveries and caesarean sections, both requiring more immediate access to health facilities. This could partially be explained by the existence of maternity waiting homes within referral health facilities in remote or conflict-prone areas, allowing women to report to the health facility well before the onset of labour. Maternity waiting homes are recommended by the World Health Organisation despite the limited (but growing) evidence available measuring their efficacy in improving maternal and neonatal outcomes in stable settings [64, 65].

### Study limitations

Caution should be used when interpreting our findings because of the uncertainty about denominators. Despite efforts by the provincial ministry of health in the Kivu to update population projections with occasional population counts, we acknowledge the risk of overreporting by household’s heads, local community leaders or even health facilities in-charges in the face of possible prospective gains such as food, money or other humanitarian commodities [44]. Population displacement is a major factor associated with armed conflict and may greatly affect the accuracy of target population estimates and reported services by health zone through the DHIS2. The size and direction of the bias is however unknown, as we were not able to obtain population displacement data from either governmental or humanitarian actors. In our analysis of health facility data, we assumed that non-reporting facilities delivered services at one-fourth the level of reporting facilities. This crude assumption may lead to over- or underestimation of coverage, but our sensitivity analysis showed that the impact of different assumptions was modest because the reporting completeness is high in most health zones. Further research however would be needed to obtain a better idea of the extent to which non-reporting is associated with facility closure or reduced service provision in conflict affected settings.

Under- or over-reporting may also affect RMNCH events reported in the DHIS2. Health workers may be tempted to under-report negative outcomes such as deaths for fear of blame or financial sanctions (for instance under performance-based financing scheme) [60, 66]. Our analysis may not be generalisable to women in need of RMNCH services who have no access to health facilities.

## Conclusions

Although good in general, the health facility reporting rate in the Kivu was negatively impacted by conflict intensity especially at the beginning of the DHIS2’s rolling-up. Routine health facility data appeared to be reliable for assessing and monitoring trends in RMNCH intervention coverage, including in health zones with high-intensity conflict. Examining whether and how the observed facility trends reflect the situation in the community will necessitate the development and testing of data collection strategies that allow the reconciliation between health facility and community-based data.

## Supplementary Information

**Additional file 1.**

## Data Availability

The data used in this study are either publicly available or can be requested directly from respective Ministries of Health.

## Abbreviations

ANC4: Fourth antenatal care visit
DHIS2: District Health Information System 2
DHS: Demographic and Health Survey
DPT: Pentavalent vaccine (Diphteria, Pertusssis, Tetanus, type b haemophilus influenza
DRC: Democratic Republic of Congo
HF: Health facility
HZ: Health zone
MICS: Multiple Indicator Cluster Survey
RMNCH: Reproductive, maternal, newborn and child health
UCDP: Uppsala Conflict Data Project Georeferenced Event Dataset
UNOCHA: United Nations Office for Coordination of Humanitarian Affairs

## Footnotes

1 0.6745 is the 0.75th quantile of the standard normal distribution. It is used as a constant in this formula to balance out the tendency of MAD to converge to the median of the half normal distribution for large N, which is the 75% percentile of a normal distribution and for which N (0.75) ~ 0.6745.

## References

[1] Wagner Z, Heft-Neal S, Bhutta ZA, Black RE, Burke M, Bendavid E (2018). Armed conflict and child mortality in Africa: a geospatial analysis. Lancet.

[2] Boerma T, Tappis H, Saad-Haddad G, Das J, Melesse DY, DeJong J, Spiegel P, Black R, Victora C, Bhutta ZA (2019). Armed conflicts and national trends in reproductive, maternal, newborn and child health in sub-Saharan Africa: what can national health surveys tell us?. BMJ Glob Health.

[3] Wagner Z, Heft-Neal S, Wise PH, Black RE, Burke M, Boerma T, Bhutta ZA, Bendavid E (2019). Women and children living in areas of armed conflict in Africa: a geospatial analysis of mortality and orphanhood. Lancet Glob Health.

[4] World Health Organization (2019). Trends in maternal mortality: 2000–2017: estimates from WHO, UNICEF, UNFPA, World Bank Group and the United Nations Population Division.

[5] UN IGME (2019). Levels and trends in child mortality.

[6] Ministere du Plan et Suivi de la Mise en Oeuvre de la Revolution de la Modernite MdlSP, MEASURE DHS, ICF International: République Démocratique du Congo Enquête Démographique et de Santé (EDS-RDC) 2013–2014. In*.*: MPSMRM, MSP, and ICF International Rockville, MD; 2014.

[7] Hogan DR, Stevens GA, Hosseinpoor AR, Boerma T (2018). Monitoring universal health coverage within the sustainable development goals: development and baseline data for an index of essential health services. Lancet Glob Health.

[8] Lindskog EE (2016). The effect of war on infant mortality in the Democratic Republic of Congo. BMC Public Health.

[9] WHO: Improving health system efficiency: Democratic Republic of the Congo: improving aid coordination in the health sector. In*.*: World Health Organization; 2015.

[10] Diese M, Kalonji A, Izale B, Villeneuve S, Kintaudi NM, Clarysse G, Ngongo N, Ntambue AM (2018). Community-based maternal, newborn, and child health surveillance: perceptions and attitudes of local stakeholders towards using mobile phone by village health volunteers in the Kenge health zone, Democratic Republic of Congo. BMC Public Health.

[11] Namazzi G, Okuga M, Tetui M, Muhumuza Kananura R, Kakaire A, Namutamba S, Mutebi A, Namusoke Kiwanuka S, Ekirapa-Kiracho E, Waiswa P (2017). Working with community health workers to improve maternal and newborn health outcomes: implementation and scale-up lessons from eastern Uganda. Global Health Action.

[12] Amouzou A, Kidanu A, Taddesse N, Silva R, Hazel E, Bryce J, Black RE (2015). Using health extension workers for monitoring child mortality in real-time: validation against household survey data in rural Ethiopia. PLoS One.

[13] Amouzou A, Banda B, Kachaka W, Joos O, Kanyuka M, Hill K, Bryce J (2014). Monitoring child mortality through community health worker reporting of births and deaths in Malawi: validation against a household mortality survey. PLoS One.

[14] Helleringer S, Arhinful D, Abuaku B, Humes M, Wilson E, Marsh A, Clermont A, Black RE, Bryce J, Amouzou A (2018). Using community-based reporting of vital events to monitor child mortality: lessons from rural Ghana. PLoS One.

[15] Cordes KM, Cookson ST, Boyd AT, Hardy C, Malik MR, Mala P, El Tahir K, Everard M, Jasiem M, Husain F (2017). Real-time surveillance in emergencies using the early warning alert and response network. Emerg Infect Dis.

[16] Boerma T (2013). Public health information needs in districts. BMC Health Serv Res.

[17] Casey SE, Mitchell KT, Amisi IM, Haliza MM, Aveledi B, Kalenga P, Austin J (2009). Use of facility assessment data to improve reproductive health service delivery in the Democratic Republic of the Congo. Confl Heal.

[18] Bhattacharya AA, Umar N, Audu A, Felix H, Allen E, Schellenberg JR, Marchant T (2019). Quality of routine facility data for monitoring priority maternal and newborn indicators in DHIS2: a case study from Gombe state, Nigeria. PloS one.

[19] Bhutta ZA (2016). Mapping the geography of child mortality: a key step in addressing disparities. Lancet Glob Health.

[20] Wise PH, Darmstadt GL (2016). The grand divergence in global child health: confronting data requirements in areas of conflict and chronic political instability. JAMA Pediatr.

[21] Victora CG, Boerma T (2018). Inequalities in child mortality: real data or modelled estimates?. Lancet Glob Health.

[22] Kadir A, Garcia DM, Romero F (2019). New ways to measure the effects of armed conflict in civilian population. Lancet Glob Health.

[23] Dialogue I-C: Political negotiations on the peace process and on transition in the DRC: global and inclusive agreement on transition in the Democratic Republic of the Congo. In: Pretoria; 2002.

[24] Stearns J (2012). North Kivu: the background to conflict in north Kivu province of eastern Congo.

[25] Vlassenroot K: South Kivu: identity, territory, and power in the eastern Congo. In: Rift Valley Institute; 2013.

[26] Weiss HF (2007). Voting for change in the DRC. J Democr.

[27] Human Rights Watch: World Report. In*.* United States of America: Human Rights Watch; 2019.

[28] Lalji N (2007). The resource curse revised: conflict and Coltan in the Congo. Harv Int Rev.

[29] UNOCHA-DRC: République Démocratique du Congo : personnes déplacées internes et retournées. In*.* Kinshasa: UNOCHA-DRC; 2020.

[30] OCHA-RDC: République démocratique du Congo: Note d’informations humanitaires pour la Province du Nord-Kivu, 26 mars 2019. In Goma: OCHA-RDC; 2019.

[31] Sundberg R, Melander E (2013). Introducing the UCDP georeferenced event dataset. J Peace Res.

[32] Croicu M, Sundberg R: UCDP GED codebook version 17.1. Department of Peace and Conflict Research, Uppsala University 2017:1–38.

[33] Marcucci G: Democratic Republic of the Congo: conflict in the eastern regions. In*.*, vol. The war report 2018. Geneva: The Geneva Academy; 2019.

[34] UNHCR (2018). Global trends: forced displacement in 2018.

[35] Wright J: Essential package of health services country snapshot: the Democratic Republic of the Congo. In*.* Bethesda, MD; 2015.

[36] District Health Information System 2 [https://www.dhis2.org/]. Accessed 26 Jan 2021.

[37] Ministère du Plan et Suivi de la Mise en œuvre de la Révolution de la Modernité (MPSMRM) MrdlSPMeI: Enquête Démographique et de Santé en République Démocratique du Congo 2013–2014. In*.* Rockville, Maryland, USA: MPSMRM, MSP et ICF International; 2014.

[38] INS: Enquête par grappes à indicateurs multiples, 2017–2018, rapport de résultats de l’enquête. Kinshasa, République Démocratique du Congo. In*.* Kinshasa; 2019.

[39] Eck K (2012). In data we trust? A comparison of UCDP GED and ACLED conflict events datasets. Cooperation Conflict.

[40] DR Congo - Health Zones [https://data.humdata.org/dataset/dr-congo-health-0]. Accessed 26 Jan 2021.

[41] Maina I, Wanjala P, Soti D, Kipruto H, Droti B, Boerma T (2017). Using health-facility data to assess subnational coverage of maternal and child health indicators, Kenya. Bull World Health Organ.

[42] WHO (2017). Data quality review: module 2: desk review of data quality.

[43] Iglewicz B, Hoaglin DC: How to detect and handle outliers, vol. 16: Asq Press; 1993.

[44] Harrell-Bond B, Voutira E, Leopold M (1992). Counting the refugees: gifts, givers, patrons and clients. J Refug Stud.

[45] Smits J, Monden CJPO: Twinning across the developing world. 2011, 6(9):e25239. 10.1371/journal.pone.0025239 PMC3182188 21980404

[46] Blencowe H, Cousens S, Jassir FB, Say L, Chou D, Mathers C, Hogan D, Shiekh S, Qureshi ZU, You DJTLGH: National, regional, and worldwide estimates of stillbirth rates in 2015, with trends from 2000: a systematic analysis. 2016, 4(2):e98-e108. 10.1016/S2214-109X(15)00275-2 26795602

[47] UN IGME: Levels and trends in child mortality: 2019 report. In: United Nations Children’s Fund; 2019.

[48] Mootz JJ, Muhanguzi FK, Panko P, Mangen PO, Wainberg ML, Pinsky I, Khoshnood K (2018). Armed conflict, alcohol misuse, decision-making, and intimate partner violence among women in northeastern Uganda: a population level study. Confl Heal.

[49] Akresh R, Caruso G, Thirumurthy H (2016). Detailed geographic information, conflict exposure, and health impacts.

[50] Akseer N, Rizvi A, Bhatti Z, Das JK, Everett K, Arur A, Chopra M, Bhutta ZA (2019). Association of Exposure to civil conflict with maternal resilience and maternal and child health and health system performance in Afghanistan. JAMA Netw Open.

[51] Congo Research Group: Congo. Forgotten: the numbers behind Africa’s longest humanitarian crisis. In: Center On International Cooperation, New York Univesity; 2019.

[52] Akhlaq A, McKinstry B, Muhammad KB, Sheikh A (2016). Barriers and facilitators to health information exchange in low-and middle-income country settings: a systematic review. Health Policy Plan.

[53] Schulman S: Bringing phone reception to a remote mountain town in the Democratic Republic of the Congo – in pictures. In: The Guardian*.* 2015.

[54] DHIS2 Android overview [https://www.dhis2.org/android]. Accessed 26 Jan 2021.

[55] Kandala N-B, Mandungu TP, Mbela K, Nzita KP, Kalambayi BB, Kayembe KP, Emina JB (2014). Child mortality in the Democratic Republic of Congo: cross-sectional evidence of the effect of geographic location and prolonged conflict from a national household survey. BMC Public Health.

[56] Chiara Altare, Espoir Bwenge Malembaka, Maphie Tosha, Christopher Hook, Hamady Ba, Stéphane Muzindusi Bikoro, Thea Scognamiglio, Hannah Tappis, Jerome Pfaffmann, Ghislain Bisimwa Balaluka et al: Health services for women, children and adolescents in conflict affected settings: experience from North and South Kivu, Democratic Republic of Congo. Conflict and health 2020 In press. 10.1186/s13031-020-00265-1 PMC7254646 32514296

[57] World Health Organization: Improving health system efficiency: Democratic Republic of the Congo: improving aid coordination in the health sector. In*.*: World Health Organization; 2015.

[58] Odhiambo J, Jeffery C, Lako R, Devkota B, Valadez JJHP, Planning: measuring health system resilience in a highly fragile nation during protracted conflict: South Sudan 2011–2015. 2019. 10.1093/heapol/czz160 PMC7152724 31876921

[59] Médecins Sans Frontières: International Activity Report 2018. In*.* Geneva, Switzerland.

[60] Maini R, Mounier-Jack S, Borghi J (2018). Performance-based financing versus improving salary payments to workers: insights from the Democratic Republic of Congo. BMJ Glob Health.

[61] RDC-PROSANIplus: Renforcement de la communication des données sanitaires et amélioration de leur qualité et utilisation grâce à l’intégration des données nationales et celles du projet dans la plateforme DHIS 2: enseignements tirés de la RDC. In*.*

[62] Mclean D: Impact of violence on medical and humanitarian services in north Kivu, DRC. In*.* Edited by Analysis Department M, Brussels; 2015.

[63] International Alert: Beyond stabilisation: understanding the conflict dynamics in north and south Kivu, Democratic Republic of Congo. In*.* London, United Kingdom; 2015.

[64] Dadi TL, Bekele BB, Kasaye HK, Nigussie TJBhsr: Role of maternity waiting homes in the reduction of maternal death and stillbirth in developing countries and its contribution for maternal death reduction in Ethiopia: a systematic review and meta-analysis 2018, 18(1):748. 10.1186/s12913-018-3559-y PMC6167854 30285757

[65] World Health Organization: WHO recommendations on health promotion interventions for maternal and newborn health 2015: World Health Organization; 2015. 26180864

[66] Turcotte-Tremblay A-M, Gali-Gali IA, De Allegri M, Ridde V (2017). The unintended consequences of community verifications for performance-based financing in Burkina Faso. Soc Sci Med.

