# Supplementary material for: The use of health facility data to assess the effects of armed conflicts on maternal and child health: experience from the Kivu, DR Congo
Source: BMC Health Serv Res. 2021 Sep 13;21(Suppl 1):195. doi: 10.1186/s12913-021-06143-7 (PMC8436447; doi:10.1186/s12913-021-06143-7)
Supplement: Supplementary file 1 — Additional file 1. [file 12913_2021_6143_MOESM1_ESM.docx]

**ADDITIONAL FILE**

**{Boerma, 2013 #216}The use of health facility data to assess the effects of armed conflicts on maternal and child: lessons from the Kivu, DR Congo**

Table of Contents

[1. Health facility managing authority 2](#_Toc41931339)

[2. Alternative conflict intensity definition 2](#_Toc41931340)

[3. Definition of indicators used in the analysis 3](#_Toc41931341)

[4. Health facility data quality assessment 4](#_Toc41931342)

[4.1. Presence of outliers 4](#_Toc41931343)

[4.2. Correcting for implausible reported numbers using monthly data 4](#_Toc41931344)

[4.3. Internal consistency within variable: overall time trends 5](#_Toc41931345)

[4.4. External consistency with related indicators 6](#_Toc41931346)

[4.5. Consistency of denominators 7](#_Toc41931347)

[5. Adjustment factor for level of reporting – sensitivity analysis 11](#_Toc41931348)

[6. Visual analysis of the difference between reported numbers during conflict and non-conflict months 12](#_Toc41931349)

[References 17](#_Toc41931350)

# Health facility managing authority

**Table S1. Health facility managing authority by conflict location**

| Health facility managing authority | Conflict intensity | | | Total |
| --- | --- | --- | --- | --- |
|  | Low | Moderate | High |  |
| Faith-based | 515 (36.1) | 92 (33.6) | 158 (41.9) | 765 (36.8) |
| Private | 226 (15.8) | 25 (9.1) | 27 (7.2) | 278 (13.4) |
| Public | 687 (48.1) | 157 (57.3) | 192 (50.9) | 1036 (49.8) |

Data are n (%).

# Alternative conflict intensity definition

We examined another possibility of classifying health zones by conflict intensity. We defined conflict intensity by health zone as the cumulative number of conflict-related fatalities divided by the average annual population between 2015-2018. We used the formula:

Cumulative death rate$=\frac{\sum_{2015}^{2018} conflict deaths}{\frac{\sum_{2015}^{2018} population}{4}} \times100,000$ (1)

We considered the distribution of deaths and classified the conflict intensity in health zones as low, moderate and high based on a cumulative death rate below 15, between 15-29.9, and at least 30 per 100,000 inhabitants respectively (Figure S1).

**Figure S1. Classification of Kivu health zones by conflict intensity, using the cumulative deaths rate over the entire study period (2015 – 2018).**


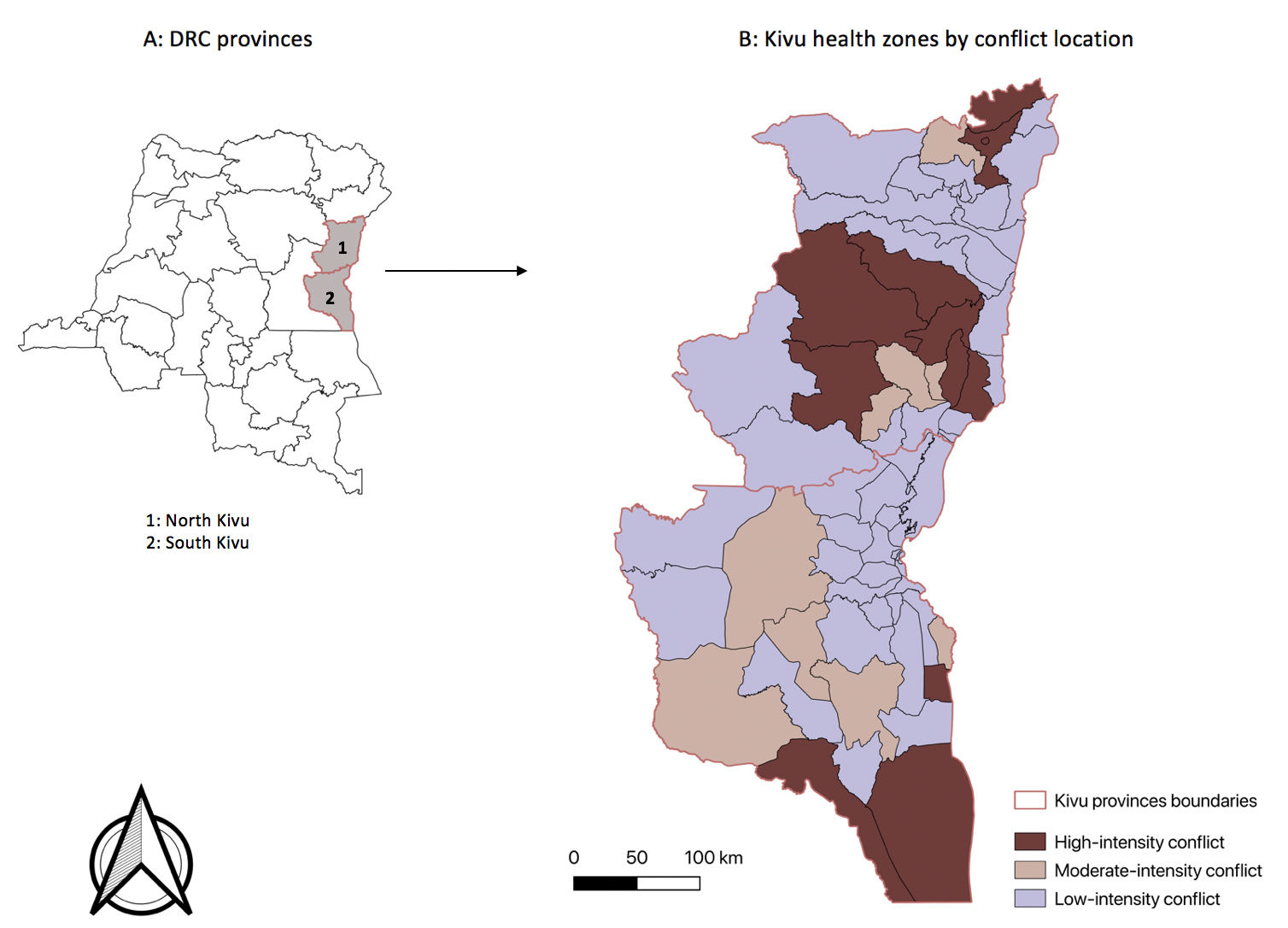


We explored other possibilities for classifying health zones using an annual average conflict death rate, quintiles of both the cumulative death rate and crude death numbers with the bottom 40% ranked as least insecure, the next 40% moderately insecure and the top 20% most insecure, and conversely, following an approach used to group wealth quintiles in some LMICs contexts [1].

The classification based on the cumulative death rate, although simple, could lead to some level of misclassification of health zones depending on the dynamic of the conflict over the years.

**Table S2. Annual distribution of health zones by conflict intensity (defined in terms of annual death rate)**

| Conflict intensity | 2015 | 2016 | 2017 | 2018 |
| --- | --- | --- | --- | --- |
| Low-intensity conflict | 57 (83.8) | 53 (77.9) | 40 (58.8) | 39 (57.4) |
| Moderate-intensity conflict | 7 (10.3) | 9 (13.2) | 8 (11.8) | 13 (19.1) |
| High-intensity conflict | 4 (5.9) | 6 (8.8) | 20 (29.4) | 16 (23.5) |

# Definition of indicators used in the analysis

We used four key service indicators along the RMNCH continuum to investigate the impact of conflict on RMNCH. They are defined as follows:

**Table S3. Definition of the study indicators**

| **Indicator** | **Definition** | **Numerator** | **Denominator** |
| --- | --- | --- | --- |
| Antenatal Care Four (ANC4) | Proportion of pregnant women who attended four antenatal visits during pregnancy by skilled health personnel | Number of women who attended four antenatal visits during pregnancy by skilled health personnel | Estimated number of pregnancies in a population |
| Institutional deliveries | Proportion of deliveries occurring in health facilities | Number of deliveries occurring in health facilities | Expected deliveries in the population |
| Caesarean sections | Proportion of facility deliveries by Caesarean section | Number of facility deliveries by Caesarean section | Total number of facility deliveries |
| Third dose of the pentavalent vaccine coverage | Percentage of infants who received the third dose of the pentavalent vaccine | Number of infants who received the third dose of the pentavalent vaccine | Estimated number of children under 12 months in the given area |

# Health facility data quality assessment

## Presence of outliers

Table S4 presents the distribution of health zones with outlying reported ANC1, DPT1 and DPT3 by conflict location.

**Table S4. Health zones (by conflict intensity) with extreme outliers**

| Indicator | 2015 | | 2016 | | 2017 | | 2018 | |
| --- | --- | --- | --- | --- | --- | --- | --- | --- |
|  | Total | N (%) | Total | N (%) | Total | N (%) | Total | N (%) |
| ANC1 |  |  |  |  |  |  |  |  |
| Low conflict | 57 | 5 (8.7) | 53 | 3 (5.6) | 40 | 3 (7.7) | 39 | 11 (29.0) |
| Moderate conflict | 7 | 0 | 9 | 0 | 8 | 3 (37.5) | 13 | 0 |
| High conflict | 4 | 0 | 6 | 1 (16.7) | 20 | 4 (20.0) | 16 | 7 (43.8) |
|  |  |  |  |  |  |  |  |  |
| DPT1 |  |  |  |  |  |  |  |  |
| Low conflict | 57 | 2 (3.5) | 53 | 2 (3.8) | 40 | 6 (15.0) | 39 | 11 (28.2) |
| Moderate conflict | 7 | 0 | 9 | 1 (11.1) | 8 | 1(12.5) | 13 | 4 (30.8) |
| High conflict | 4 | 0 | 6 | 0 | 20 | 4 (20.0) | 16 | 5 (31.3)) |
|  |  |  |  |  |  |  |  |  |
| DPT3 |  |  |  |  |  |  |  |  |
| Low conflict | 57 | 0 | 53 | 0 | 40 | 0 | 39 | 0 |
| Moderate conflict | 7 | 0 | 9 | 0 | 8 | 0 | 13 | 0 |
| High conflict (n=12) | 4 | 0 | 6 | 0 | 20 | 0 | 16 | 0 |
|  |  |  |  |  |  |  |  |  |

Data are n and n (%). Classification of health zones by conflict location is based on the annual conflict death rate.

## Calculating expected numbers and correcting for implausible reported numbers using monthly data

In conflict settings, the provision of RMNCH services may significantly fluctuate over time as a result of conflict and subsequent population displacement. That makes the correction effort difficult and less straightforward. We adopted a conservative approach whereby an extreme outlier (modified Z >5) was considered implausible and corrected if it was three times greater than the expected number. The process started by calculating the median value of each RMNCH indicator for the entire time series (48 months). We considered this number as the expected value for the middle month of the time series (January 2017). We then computed a monthly growth rate as the 12^th^ of 3% of the central value. The growth rate was added to and subtracted from the expected middle number to incrementally estimate expected values for the following and previous months, respectively. The correction consisted in replacing the reported with the expected number and was done for four DPT observations (zone-months) and one facility delivery zone-month.

## Internal consistency within variable: overall time trends

Reporting consistency over time was examined by assessing the ratio between reported number of a given variable in 2018 over the mean reported number between 2015-2017. Inconsistent reporting is observed if the reported number for 2018 falls beyond ±33% of the mean value for the period 2015-2017, that is, if the ratio between the number reported in 2018 over the mean reported number for 2015-2017 surpasses 1±.33. For the Kivu, reporting consistency was relatively good. Seven health zones (10.3%) inconsistently reported for ANC1 (with a ratio for inconsistent health zones varying between 1.34–1.72), 25 (36.8%) for ANC4 (one health zone with a ratio of 0.63, and 24 health zones with the ratio between 1.33 and 2.82), 4 (5.9%) for DPT1 (ratio ranging between 1.34–1.72), 6 (8.8%) for DPT3 (the ratio for inconsistent health zones: 1.34-1.70), 11 (16.2%) for measles (the ratio for inconsistent health zones varying between 1.34–1.81), 21 (30.8%) health zones for OPD (with one health zone with ratio of 0.58 and 20 health zones with a ratio varying between 1.33 and 2.27).

**Table S5. Distribution of health zones with inconsistent reporting over time, by conflict location**

| Indicator | Low insecurity (n=47) | Moderate insecurity (n=9) | High insecurity (n=12) | Total (n=68) |
| --- | --- | --- | --- | --- |
| ANC1 | 3 (6.4) | 1 (11.1) | 3 (25.0) | 7 (10.3) |
|  |  |  |  |  |
| ANC 4 | 17 (36.2) | 4 (44.4) | 4 (33.3) | 25 (36.8) |
|  |  |  |  |  |
| DPT1 | 3 (6.4) | 0 (0) | 1 (8.3) | 4 (5.9) |
|  |  |  |  |  |
| DPT3 | 4 (8.5) | 1 (11.1) | 1 (8.3) | 6 (8.8) |
|  |  |  |  |  |
| Measles | 7 (14.9) | 2 (22.2) | 2 (16.7) | 11 (16.2) |

Data are number (percentage) of health zones with inconsistency in reporting over time. Conflict location is based on the cumulative conflict death rate for the period 2015-2018.

## External consistency with related indicators

We examined the absolute difference between the expected and reported ANC1/DPT1 and DT1/DPT3 ratios. The average expected ratios in the Kivu were calculated from the average of coverage values for North Kivu and South Kivu as reported in the 2017-2018 DRC MICS. We then calculated the difference between the ratios of numbers of ANC1/DPT1 and DPT1/DPT3 reported in the DHIS2 and the expected values. We decided to regard a ratio difference below 5 as an indication of good quality, values between 5-14.9 indicated moderate quality (moderate inconsistencies between expected and observed values) and values equal to or higher than 15 indicated poor quality [2]. Overall, the median (interquartile range) of the ratio ANC1/DPT1 and DPT1/DPT3 was 10.0 (6.2–16.7) and 12.0 (10–13.4) respectively. There appeared to be a better consistency between DP1-DPT3 than between ANC1-DPT1, but more noticeably in insecure zones. About two-third (66.2%, n=45) of the health zones appeared to have a good to moderate ANC1-DPT1 consistency against up to 91.2% (n=62) for DPT1-DPT3.

**Table S6. External consistency between ANC1-DPT1 and DPT1-DPT3 by conflict location (based on the cumulative conflict death rate)**

|  | Low insecurity (n=47) | Moderate insecurity (n=9) | High insecurity (n=12) | Total |
| --- | --- | --- | --- | --- |
| Consistency ANC1-DPT1 |  |  |  | 10.0 (6.2–16.7) |
| Good | 9 (19.2) | 0 (0) | 2 (16.7) | 11 (16.2) |
| Moderate | 25 (53.2) | 6 (66.7) | 3 (25.0) | 34 (50.0) |
| Low | 13 (27.7) | 3 (33.3) | 7 (58.3) | 23 (33.8) |
|  |  |  |  |  |
| Consistency DPT1-DPT3 |  |  |  | 12.0 (10–13.4) |
| Good | 1 (2.1) | 0 (0) | 0 (0) | 1 (1.5) |
| Moderate | 41 (87.2) | 9 (100) | 11 (91.7) | 61 (89.7) |
| Low | 5 (10.6) | 0 (0.0) | 1 (8.3) | 6 (8.8) |
| *: Data are n (%) of health zones falling in each category and median (interquartile rage) of the difference between expected and reported ratios. Conflict location is based on the cumulative conflict death rate for the period 2015-2018. Consistency of denominators | | | | |

To examine the consistency of denominators, we calculated the relative difference between the expected number based on the 2017-2018 MICS and reported denominator numbers as a relative percent of the expected number of ANC1 visits and first dose of the DPT vaccine (DPT1). The formulas used are presented in Table S7. We computed the target number of pregnancies using the reported ANC1 number. The number of infants targeted for immunization was derived from the reported DPT1 number. The relative differences between the expected number and reported denominator numbers are computed as a relative percent of the expected number of ANC1 visits and DPT (Table S8).

**Table S7. Formula for assessment of consistency of denominators**

| Parameter | Formula |
| --- | --- |
| N _pregnant women, reported_ | N_ANC1, reported_ / (ANC1 coverage _survey_) |
| N _Infants, reported_ | N _DPT1, reported_ / (DPT1 coverage _survey_) |
| Difference ANC1 | (\|(N _pregnancies, reported_ - N _pregnancies, projected_)\|/ N _pregnancies, projected_ * 100% |
| Difference DPT1 | \|(N _infants, reported_ - N _infants, projected_ )\|/  N _infants, projected_ * 100% |

In DRC, the latest population census dates back to 1984, making it difficult for defining accurate target populations for health interventions. We obtained the total population estimates for health zones from the DHIS2. To obtain the population projections, we applied the crude birth rate reported in the 2017-2018 MICS (37.5 and d 46.0 live births for 1000 population in urban and rural areas respectively[3]) to the total population estimates to calculate the annual number of births. We also assumed a proportion of 2% for stillbirths, 2% for twins, 5% for abortion and 2.8% for infants who died before 6 weeks of life to estimate the number of live births, deliveries, and pregnancies.

**Table S8. Consistency of denominators for the Kivu**

| Parameter | 2015 | 2016 | 2017 | 2018 |
| --- | --- | --- | --- | --- |
| North-Kivu |  |  |  |  |
| Reported pregnancies | 331231 | 322888 | 344947 | 345613 |
| Projected Pregnancies | 372971 | 406687 | 393876 | 415957 |
| N infants (DPT1), reported | 362628 | 367877 | 401068 | 408513 |
| N infants (DPT1), projected | 337669 | 368194 | 312297 | 312901 |
| Difference ANC1 | -11.19 | -20.61 | -12.42 | -16.91 |
| Difference DPT1 | 7.39 | -0.09 | 28.42 | 30.56 |
| Difference ANC1-DPT1, reported | -9.48 | -13.93 | -16.27 | -18.20 |
|  |  |  |  |  |
| South Kivu |  |  |  |  |
| Reported pregnancies | 270748 | 291243 | 319851 | 319861 |
| Projected Pregnancies | 295952 | 304739 | 324595 | 332318 |
| N infants (DPT1), reported | 258253 | 289118 | 319474 | 323848 |
| N infants (DPT1), projected | 273282 | 281396 | 295351 | 295360 |
| Difference ANC1 | -8.52 | -4.43 | -1.46 | -3.75 |
| Difference DPT1 | -5.50 | 2.74 | 8.17 | 9.65 |
| Difference ANC1-DPT1, reported | 4.62 | 0.73 | 0.12 | -1.25 |
|  |  |  |  |  |
| Kivu |  |  |  |  |
| Reported pregnancies | 601979 | 614131 | 664798 | 665474 |
| Projected Pregnancies | 668923 | 711426 | 718471 | 748275 |
| N infants (DPT1), reported | 620881 | 656995 | 720542 | 732361 |
| N infants (DPT1), projected | 610951 | 649590 | 607648 | 608261 |
| Difference ANC1 | -10.01 | -13.68 | -7.47 | -11.07 |
| Difference DPT1 | 1.63 | 1.14 | 18.58 | 20.40 |
| Difference ANC1-DPT1, reported | -3.14 | -6.98 | -8.39 | -10.05 |

We also graphically examined the consistency of reported ANC1 and DPT1 over time, as well as the consistency between reported ANC1 and DPT1, with scatter plots.

**Figure S2. ANC1 consistency over time**

**Figure S3. DPT1 consistency over time**

**Figure S4. Consistency between ANC1 and DPT1 over time**

# Adjustment factor for level of reporting – sensitivity analysis

**Figure S5. Level and trends in fourth antenatal care visits, health facility deliveries, caesarean section rate and pentavalent vaccine by conflict intensity level, assuming k=0**


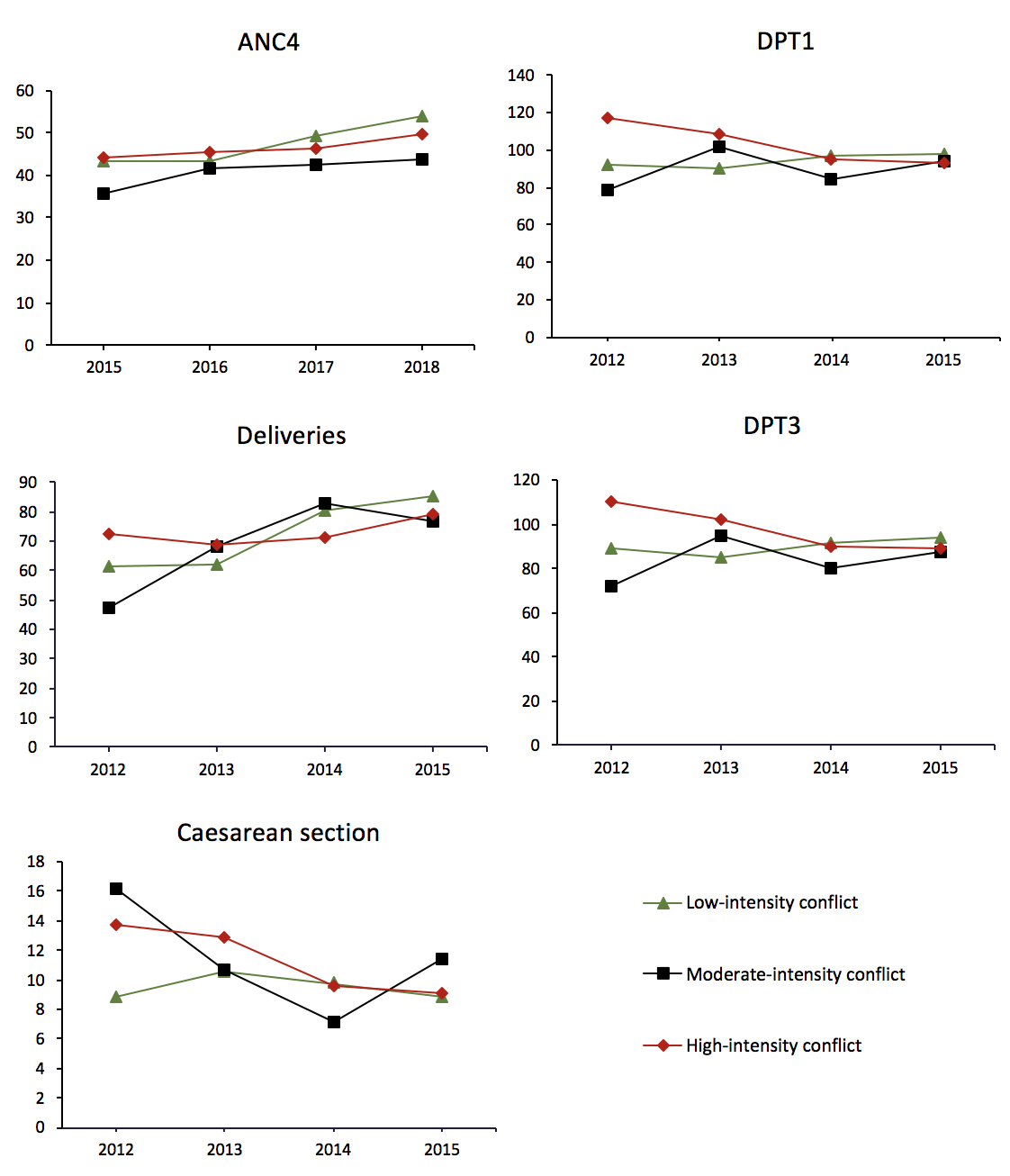


**Figure S6. Level and trends in fourth antenatal care visits, health facility deliveries, caesarean section rate and pentavalent vaccine by conflict level, assuming k=1**


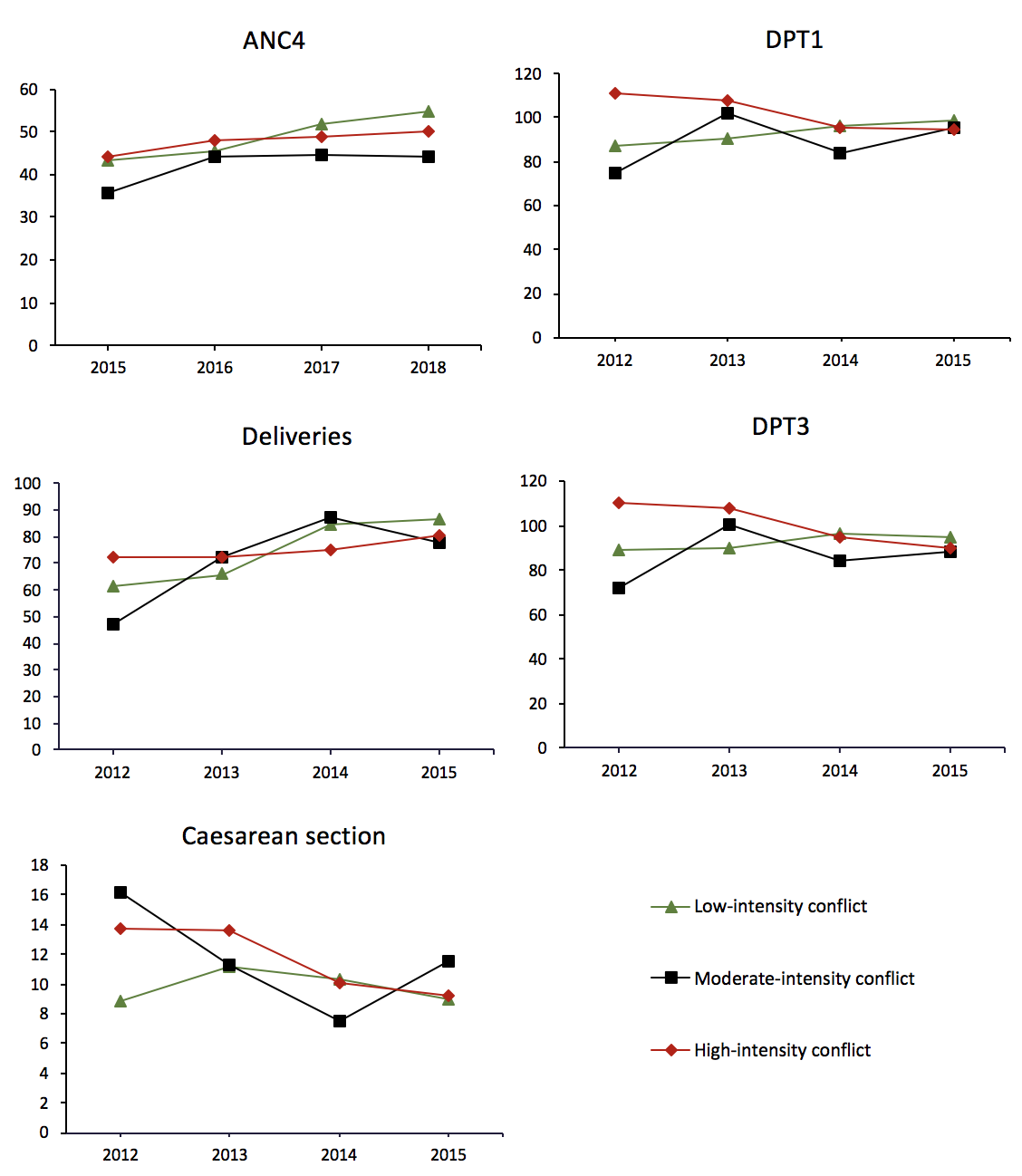


# Visual analysis of the difference between reported numbers during conflict and non-conflict months

We graphically examined the effect of conflict on reporting numbers in a sample six health zones selected of their high conflict intensity. We calculated the average number of the health indicators for all the health zones with mild or no conflict, that is, a monthly conflict fatality rate below 5 deaths per 100,000 population. We considered this average as the expected number for the middle month (January 2017) of the entire time series. We also calculated a monthly growth rate as the 12^th^ of 3% of the reported number. The growth rate was added to and subtracted from the expected central number to estimate expected value for the following and previous months, respectively. This process was repeated for each of the four indicators.

**Figure S7. Scatter plot of reported and expected number for the fourth antenatal care visit**

**Figure S8. Scatter plot of reported and expected number for Caesarean section deliveries**

**Figure S9. Scatter plot of reported and expected number for institutional deliveries**

**Figure S10. Scatter plot of reported and expected number for the third dose of pentavalent vaccine**

**Table S10. One-month lag effects of conflict intensity and insecurity on RMCH service provision in the Kivu**

|  | Threshold 1 | | Threshold 2 | | Threshold 3 | |
| --- | --- | --- | --- | --- | --- | --- |
| Difference, reported-expected numbers | Coefficient (95% CI) | P value | Coefficient (95% CI) | P value | Coefficient (95% CI) | P value |
| ANC4 |  |  |  |  |  |  |
| Severe conflict | 2.47 (-1.01; 5.95) | 0.162 | 2.17 (-1.16; 5.51) | 0.198 | 1.99 (-1.50; 5.47) | 0.259 |
| Insecurity | 2.00 (-0.89; 4.89) | 0.173 | 1.88 (-1.42; (5.18) | 0.259 | -0.64 (-4.29; 3.02) | 0.728 |
|  |  |  |  |  |  |  |
| Facility deliveries |  |  |  |  |  |  |
| Severe conflict | 2.28 (-3.82; 8.38) | 0.459 | 2.59 (-4.27; 9.45) | 0.453 | 2.43 (-4;13; 8.98) | 0.462 |
| Insecurity | 2.89 (-2.61; 8.40) | 0.298 | 1.44 (-4.62; 7.49) | 0.637 | 2.73 (-0.79; 13.36) | 0.610 |
|  |  |  |  |  |  |  |
| Caesarean section |  |  |  |  |  |  |
| Severe conflict | 0.11 (-0.46; 0.69) | 0.692 | -0.13 (-0.72; 0.47) | 0.675 | -0.21 (-0.87; 0.44) | 0.518 |
| Insecurity | 0.10 (-0.42; 0.61) | 0.704 | 0.21 (-0.30; 0.72) | 0.416 | -0.35 (-1.03; 0.34) | 0.313 |
|  |  |  |  |  |  |  |
| DPT3 |  |  |  |  |  |  |
| Severe conflict | 1.13 (-1.28, 3.53) | 0.353 | 0.95 (-1.95; 3.85) | 0.515 | 0.57 (-2.53; 3.67) | 0.715 |
| Insecurity | 1.07 (-1.01; 3.16) | 0.308 | 0.83 (-167; 3.34) | 0.509 | -2.53 (-6.57; 1.52) | 0.217 |

Thresholds 1, 2 and 3 defined respectively as: monthly death rate higher than 5, 10, 15 per 100,000 population (severe conflict); conflict event-days higher than 1, 5, 10 per 100,000 (insecurity).

# References

1. Filmer D, Pritchett LH: **Estimating wealth effects without expenditure data—or tears: an application to educational enrollments in states of India**. *Demography* 2001, **38**(1):115-132.

2. Maïga A, Jiwani SS, Mutua MK, Porth TA, Taylor CM, Asiki G, Melesse DY, Day C, Strong KL, Faye CM: **Generating statistics from health facility data: the state of routine health information systems in Eastern and Southern Africa**. *BMJ global health* 2019, **4**(5):e001849.

3. INS: **Enquête par grappes à indicateurs multiples, 2017-2018, rapport de résultats de l’enquête. Kinshasa, République Démocratique du Congo**. In*.* Kinshasa; 2019.
